# Postnatal myocardium remodelling generates inhomogeneity in the architecture of the ventricular mass

**DOI:** 10.1007/s00276-017-1945-5

**Published:** 2017-11-28

**Authors:** Pierre-Simon Jouk, Ba Luu Truong, Gabrielle Michalowicz, Yves Usson

**Affiliations:** 1 Equipe DyCTim, Laboratoire TIMC-IMAG, 38706 La Tronche cedex, France; 2 Service de génétique - CHU Grenoble Alpes, 38043 Grenoble cedex 9, France; 3 Cardiovascular Unit, Children’s Hospital 2, Ho Chi Minh City, Vietnam

**Keywords:** Remodelling, Ventricular anatomy, 3D methods, Perinatal adaptation

## Abstract

**Background:**

The 3D architecture of the ventricular mass is poorly known, although in vivo imaging techniques show the physiological inhomogeneity of ventricular walls mechanics. Polarized light imaging makes it possible to quantitatively analyse the myosin filament orientation.

**Aims:**

In this paper, we focus on the study the 3D architecture and regional isotropy of myocardial cells.

**Methods:**

Twenty normal human hearts, 10 from the perinatal period and 10 from the post-neonatal period were studied by polarized light microscopy. In each voxel of the ventricular mass (90 × 90 × 500 µm) the principal orientation segment was automatically and unambiguously extracted as well as a regional isotropy index (regional orientation tensor of the voxel neighbourhood).

**Results:**

During the first months of postnatal age, the median regional isotropy values decreased in the ventricular mesh. This global decrease was not homogeneous across the ventricular walls. From the perinatal to the neonatal period, this decrease was more marked in the inner two-third of the lateral left ventricular wall and in the right part of the interventricular septum. There was a progressive post-neonatal appearance of a particularly inhomogeneous secondary arrangement of myocardial cells with alternation of thick low-RI and thin high-RI areas.

**Conclusions:**

This study has shown a postnatal change in ventricular myocardial architecture, which became more inhomogeneous. The cell rearrangements responsible for the inhomogeneity in ventricular myocardial architecture are revealed by a variation of the regional isotropy index. These major changes are probably an adaptive consequence of the major haemodynamic changes occurring after birth during the neonatal period that generates major parietal stress variations and parietal remodelling.

**Electronic supplementary material:**

The online version of this article (10.1007/s00276-017-1945-5) contains supplementary material, which is available to authorized users.

## Background

Ventricular myocardium is a complex three-dimensional mesh of cardiomyocytes supported within a matrix of connective tissue, but the organization of this mesh at the ventricular level continues to be debated. Difficulties are numerous: the ventricular mesh has an inhomogeneous branching architecture, the heart is a multi-tissue organ made of myocardial, conjunctive and vascular cells, with multiple levels of organization. It became clear that there are no discrete structures in the ventricular mass but the inhomogeneity of cell arrangements is still unsettled [7, 22]. There are also conceptual issues leading to ambiguous nomenclature. There is also a problem of scaling: during morphogenesis, both time and size matter. Therefore, data provided in rodent studies cannot be extrapolated to large mammals and humans. Technically, it is difficult to extract the information on the 3D orientation of myocardial cells, this has been done by: anatomical dissection, microscopy, echocardiography and MRI. Each Technique gave results that are not easy to conciliate [1, 2, 5, 6, 13, 23].

As for any complex structure, it must be studied by breaking it down into simpler components, while attempting not to lose the overall picture or at least not overstating the results obtained from a single component or subregion. In this study, we focus on the principal orientation of the myosin filaments measured by polarized light imaging (PLI), which provides a robust measurement of the orientation of cardiomyocytes [12].

While studying the 3D arrangement of myocardial cells during foetal life and postnatal life with PLI, we observed major 3D rearrangements of cardiomyocytes that occur during postnatal cardiovascular adaptation [10, 11]. In previous articles, we concluded that to advance the description of ventricular mesh organization, the following question ought to be addressed: is it possible to characterize rapid variations in orientation between cardiomyocytes within the entire ventricular mass area? However, because our techniques were limited at that time (measurement range limited to a quarter of a sphere) we could not draw solid conclusions. The technical development of our PLI technique has now been completed and the principal orientation is measured unambiguously over a semi-sphere domain with high resolution in each voxel of the entire ventricular mass [4]. This made it possible to analyse the 3D regional isotropy and its variation during postnatal development.

The aim of this study was to describe the modifications of the 3D cardiomyocyte orientation of the ventricular mass during perinatal and post-neonatal period.

## Materials and methods

### Ethics statement

Grenoble University Hospital (GUH) owns a legally declared collection of embedded tissue sections collected after autopsy for perinatal and infant death performed for a diagnostic purpose. Written consent was obtained from the parents or guardians at the time of the request for autopsy authorization and for research authorization on normal and abnormal development. The research protocol has been approved by the institutional review board of GUH. Samples dedicated to research purposes were kept anonymous. The study was conducted in accordance with the 1964 Declaration of Helsinki and its later amendments.

### Materials

Twenty hearts of eutrophic newborns and infants, without detectable cardiac anomalies, were studied. There were 10 perinatal hearts (7 stillbirths and 3 early neonatal deaths) and ten post-neonatal deaths at 8–43 weeks.

### Histological preparation and polarized light imaging

**Fig. 1:**
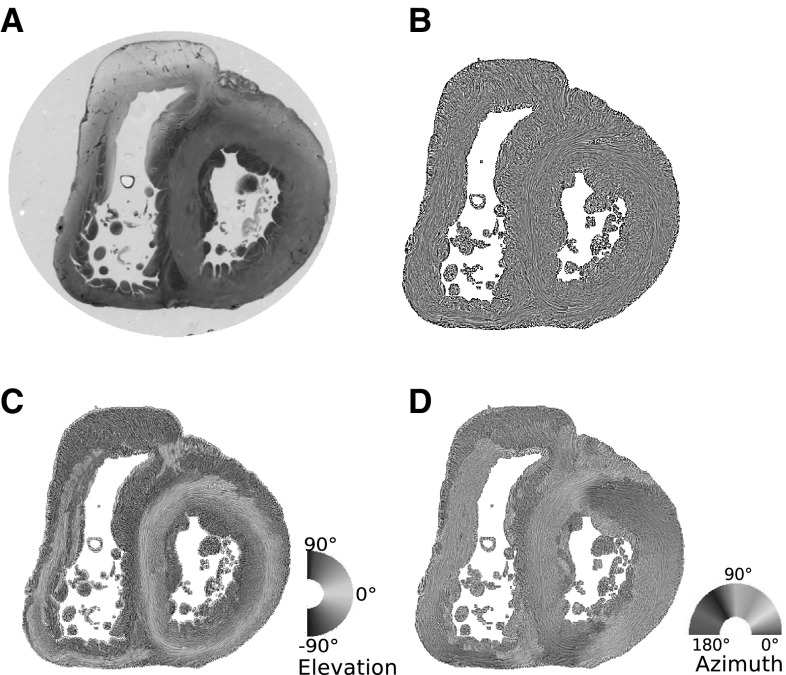
Illustration of the different maps obtained by PLI. **a** Short-axis section (thickness, 500 µm) made after resin embedding and viewed in transmitted light. **b** Same section, orientation map in streamline representation, LIC texture. **c** False colour elevation map with superimposition of LIC texture (false colour scale from − 90° to + 90°). **d** False colour azimuth map of the section with superimposition of LIC texture (false colour scale from 0° to 180°)

The protocol is the same as reported in previous publications [8, 11], as briefly summarized hereafter (Fig. 1). Heart samples were fixed in a solution of 4% neutral buffered formaldehyde. The ventricular mass was removed by cutting the atriums 1 mm above the atrioventricular groove and the great arteries 3 mm from the semi-lunar valves. The ventricular masses were then embedded in methyl methacrylate (MMA). Before sectioning, three fiducial markers were drilled perpendicular to the coronal plane on the sides of the poly-MMA resin block. A series of 500-μm thick sections were cut with a diamond wire saw (ESCIL^R^) along the heart short axis. Due to the thickness of the wire saw, 500 µm were lost and consequently, the section spacing was 1 mm. The use of MMA made it possible to cancel the form birefringence of collagen because MMA and collagen refraction indices matched well. We previously demonstrated that in this condition the birefringence of the myocardium is essentially due to the crystalline uniaxial positive birefringence of myosin. The orientation information can then be extracted with the classical PLI techniques on a three-axis rotation stage adapted to biology, particularly to the low value of birefringence and the size of the samples. This allows extracting the voxel principal orientation that is the mean orientation of all myosin filaments contained in a voxel (90 × 90 × 500 μm^3^). Such a voxel contains around 500 myocardial cells. The orientation information is expressed by two angles: the azimuth angle, that is the angle between the east–west axis of the optical bench stage and the projection of the voxel principal orientation on the stage plane (0°–180°); and the elevation angle, that is the angle corresponding to the obliquity of the voxel principal orientation with respect to the plane of the section, in other words it is the way the voxel principal orientation escapes from the section plane (− 90° to + 90°). Both these angles are defined in an absolute 3D Cartesian coordinate system. Registration of the successive sections was obtained by aligning the fiducial markers.

### Statistical methods

#### Measurement of the regional isotropy index (RI)

**Fig. 2:**
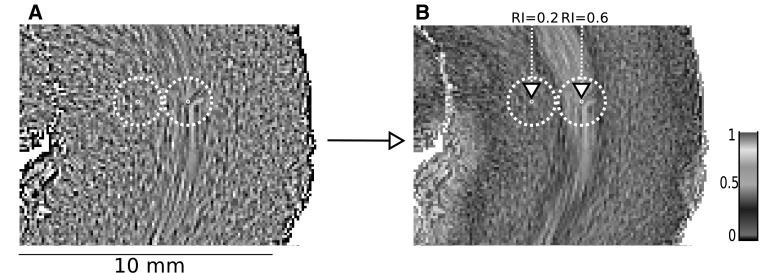
Representation of regional index (RI). **a** Part of the lateral wall of the left ventricle of a 12-weeks post-natal heart, LIC texture. The two white centred dashed circles represent the intersection of a 1 mm radius sphere with the section plane. The ellipsoid of the orientation tensor is calculated on the set of voxels intersecting the surface of this 1 mm radius sphere. From this tensor is calculated the RI index that is allocated to the voxel center of the sphere (white point). **b** The result of RI map of the same 2 centred spheres: on the left, the central pixel has a low RI index: 0.2 (neighbourhood pixels have nearly similar orientation as the centre pixel) and on the right the central pixel has a high RI index: 0.6 (almost every orientation of neighbourhood voxels are represented)

A characterization of the orientation variations between cardiomyocytes within the entire ventricular mass area must respond to 2 main criteria: it must be automated, without any manual operator intervention and it must be rotationally invariant: no orientation in the heart is to be favoured. The regional isotropy index we computed responds to these criteria. For this purpose, the principal orientation of each voxel has to be compared with the principal orientations of the 150 neighbourhood voxels situated on the surface of the 1 mm radius sphere centred on the studied voxel. Thus, a matrix of 150 rows and 3 columns is obtained. Each row contains the triplet of orientation coordinates of the 150 neighbourhood voxels. A symmetric inertia matrix (3 by 3) called tensor of orientations is built from the product of the previous matrix by its transpose. Diagonalization of the resulting inertia matrix provides its eigenvalues (*λ*1, *λ*2, *λ*3), which are the diameters of the scattering ellipsoid of the neighbourhood orientations (Fig. 2).

The RI index was calculated as follows:

$${\text{RI}} = 1.5{\text{ }}{{\left( {\lambda _{2} + \lambda _{3} } \right)} \mathord{\left/ {\vphantom {{\left( {\lambda _{2} + \lambda _{3} } \right)} {(\lambda _{1} + \lambda _{2} + \lambda _{3} )}}} \right. \kern-\nulldelimiterspace} {(\lambda _{1} + \lambda _{2} + \lambda _{3} )}}\;{\text{with all }}\lambda _{i}> 0$$

RI varies from 0 to 1, that is 0 for complete anisotropy (*λ*_1_ > > *λ*_2_ + *λ*_3_), every neighbourhood voxels have the same orientation as the centre voxel, and 1 for pure isotropy (*λ*_1_ = *λ*_2_ = *λ*_3_), all possible orientations are observed, meaning the variation of orientation is maximal between centre and neighbourhood.

It must be noted that this RI index must be differentiated from fractional anisotropy (FA) investigated by diffusion tensor MRI, where *λ*_1_, *λ*_2_ and *λ*_3_ are the eigenvalues of the diffusion ellipsoid of water in each voxel studied. RI refers to an orientation tensor and MRI FA to an ellipsoid of water diffusion [18].

Quantitative studies of the variation of RI during perinatal and post-neonatal development were conducted on each of the 20 hearts studied while analysing the distribution of RI in three consecutive sections of the mid-ventricular mass. The RI distributions were shown with box and whisker plots. The relationships between growth (ventricular weight or developmental age) and RI were investigated with classical linear regression analysis and tested with the Spearman test.

The linear transmural plot profile of RI was also studied across the left ventricular free wall and the interventricular septum at the equatorial level. Thickness of the measurement line was 5 pixels for averaging purpose. The statistical differences between the minimum and the maximum transmural values of RI in the perinatal and the post-neonatal hearts were investigated by Student-test.

### Visualization

The orientation measurements were summarized with false colour maps encoding for the values of azimuth angles, elevation angles and RI values. These maps share a common Cartesian reference system such that the inferior side of the ventricular mass is set parallel to a horizontal axis (east–west axis of the optical stage during the acquisition phase). There is a strict mathematical correspondence between the Cartesian coordinate system (*x, y, z*) and the more frequently used cylindrical or semi-spheroidal coordinate system that defines the principal orientation by the helical and imbrication angles. Cylindrical and semi-spheroidal coordinate system have clear advantages for visualization when studying structures with approximate rotational symmetry, the left ventricle for example. As we study the whole ventricular mass, a complex geometrical structure with 2 cavities and highly variable curvature of the walls and interventricular septum (IVS), we choose the 3D Cartesian coordinate system for visualization because it does not require to define a symmetry axis. It is also required for the RI index calculations. To provide readable and comprehensive information on myocardial cell orientations, we also used a streamline representation based on a classical Line Integral Convolution (LIC) algorithm [3, 21]. It creates a texture image built from limited regional tractographies (5 voxels span). This LIC texture can be superimposed on the azimuth, elevation and RI maps (Fig. 1).

## Results

This variation was studied while comparing the 10 perinatal hearts to the 10 post-neonatal hearts. For analysis of the linear regression, we chose ventricle weight, a physical measurement, as the main developmental marker because it is less biased than age, with the value taking into account an accurate duration (postnatal age) but which is actually preceded by a variable gestational duration that can only be approximated.

**Fig. 3:**
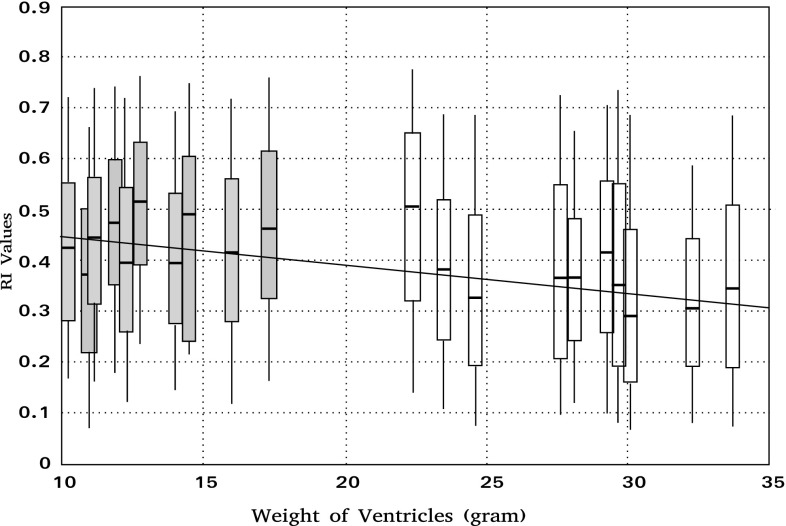
Linear regression analysis of the median of RI value distribution according to ventricular weight of 20 stillbirth and postnatal hearts. The distribution of RI has been calculated for each heart in three equatorial sections and is shown as “box and whisker” plots (lower whisker, box lower edge, box centre, box upper edge, upper whisker corresponding to 5th, 25th, 50th, 75th and 95th percentiles, respectively; perinatal hearts in grey; post-neonatal hearts in white). Regression analysis shows a statistically significant negative link between the RI median and ventricular weight

There is a progressive decrease in RI values during the 1st year of postnatal life (Fig. 3). Linear regression analyses of the RI distribution median depending on ventricular weight and age showed significant negative correlations (weight, *r* = − 0.748, *p* = 0.0008). This correlation is also statistically significant with age but lesser (*r* = − 0.567, *p* = 0.02), as could be expected because of the lack of robustness of the estimated gestational duration.

**Fig. 4:**
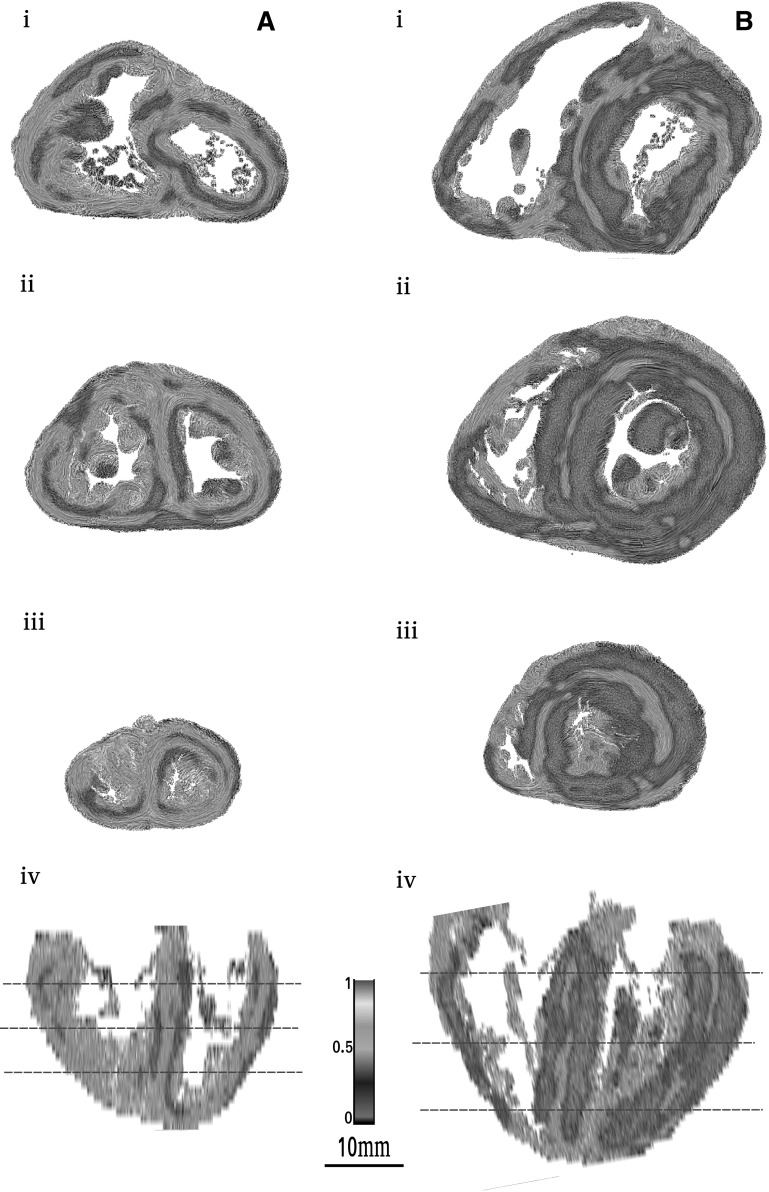
Regional isotropy maps superimposed with LIC texture of two hearts: stillbirth (**a**) and 12 weeks of age (**b**). *i* Basal section, *ii* mid-section, *iii* apical section, *iv* reconstructed long horizontal long-axis RI map, red dotted lines of the *i, ii* and *iii* section levels. The combined examination of the short-axis section levels and the horizontal long-axis section shows that the RI areas extend through the ventricular mass. The comparison of the two columns (**a, b**) confirms a general thinning tendency of the high RI layers and conversely broadening of the low-RI layers

**Fig. 5:**
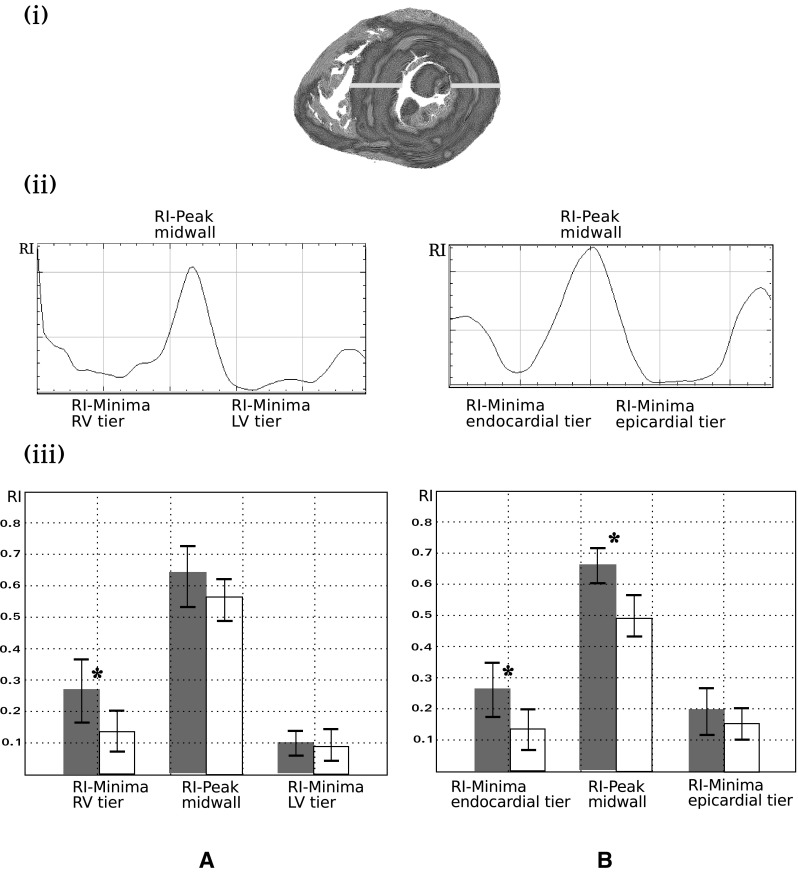
Comparative histograms of mean RI values in the IVS (**a**) and the left ventricular wall (**b**), in the 2 groups: perinatal (grey) and post-neonatal (empty boxes). *i* RI map of an equatorial section of one of the studied heart (12 weeks of age), the yellow rectangles indicate the region of interest (ROI) in the IVS and left ventricular wall for determination of the RI plot profiles, *ii* plot profile with “W” pattern in the IVS (A) and left ventricular wall (**b**), *iii* histograms of mean RI values and standard deviation in the IVS (**a**) the left ventricular wall (**b**) and in 2 groups (10 perinatal hearts in grey and 10 post-neonatal hearts empty boxes). From right side to left side of IVS, the averaged RI minima RV tier is higher in the perinatal group than in the postnatal group (*p* < 0.05). From endocardium to epicardium at the left LVW level, the RI-Minima endocardial tier and the RI-Peak midwall is lower in the post-neonatal group than in the perinatal group (*p* < 0.01; *p* < 001 respectively). *Statistically significant differences (*p* value < 0.05)

Visually, the combined examination of the short-axis and horizontal long-axis sections (Fig. 4) shows that the relative disposition of the areas of low- and high-RI values through the ventricular mass changes depending on age. The low RI areas became prominent in the left ventricular wall and the interventricular septum (IVS) in the post-neonatal period. However, a thin area of high RI persisted in the middle of these low-RI areas, except at the inferior wall of the left ventricle. Full data of two hearts are available in the online data supplements: Video 1A and 1B (stillbirth); Video 2A and 2B (12 weeks postnatal age). This decreasing trend of RI varies across the ventricular walls. Transmural plot profile of RI shows a general “W” shaped pattern with a midwall high RI peaks surrounded by 2 low RI minimas. From the perinatal to the neonatal period, the RI decrease is more marked in the inner two-third of the lateral left ventricular wall and in the right part of the interventricular septum. The transmural gradient between RI-minima and RI peak increases only between midwall and subendocardial tier of the LVW (Fig. 5).

**Fig. 6:**
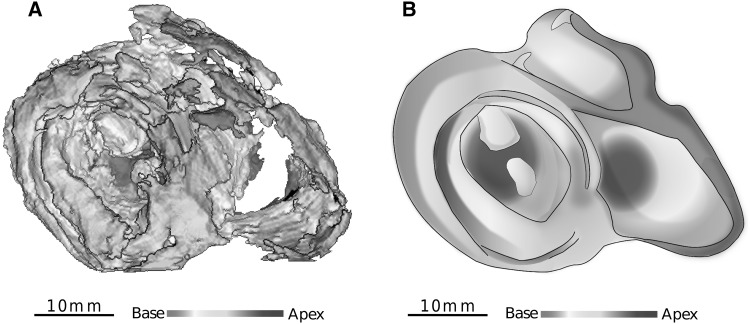
3D views of the low-RI layers in a 12-week postnatal heart and artist drawing summarizing the organization pattern. **a** Ventricular areas with an RI index < 0.3 were segmented and reconstructed with the 3D viewer. False colour scale (red to deep blue) is used to encode the axial level from base to apex. **b** The artistic view emphasizes the representation of the inhomogeneous pattern sustained by the study of RI distribution in post-neonatal life. The same false colour coding than in A is used for baso-apical representation. The high RI areas are not directly viewed but inferred from the vacuum between low-RI areas, for representation they are shadowed in grey. The high RI area of the IVS is not visible in A because it is hidden by superficial subepicardial basal low RI areas but is still represented in B for summarizing (refers to Fig. 4iv)

The low-RI index layers smaller than 0.3 (deep blue to purple false colours) were analysed within the ventricular mass and two stages are illustrated in Fig. 4. The patterns observed vary according to postnatal age. In perinatal hearts, low-RI layers are thin, the IVS is planar. In post-neonatal hearts, the low-RI layers become thicker, the IVS bulges toward the right ventricle, the right ventricular infundibulum projects over the anterior part of the left ventricle, and the layers lying near the inferior side of the right ventricle disappear at the basal tier. The relationships between low and high RI layers through the whole ventricular mass can be visualized in 3D viewed from the base (Fig. 6).

## Discussion

There are strong relationships between the vector field of cardiomyocytes orientation, as represented by line integral convolution, and the force field resulting from cardiomyocytes contraction. Thus, the post-natal remodelling of myocardial cells network is correlated with postnatal modifications of ventricular wall motions.

The RI index decreases from perinatal to post-neonatal period, from an isotropic towards a more anisotropic organization. But this global trend is not homogeneous across the ventricular walls, the mid RI peak in the LVW and the minimum RI in the right part of the IVS decrease significantly more than in the other areas. We have already demonstrated [10] that the perinatal highly isotropic state is compatible with the quasi-geodesical pattern already suggested by Krehl, Streeter and Peskin [17, 22]. The smooth variation of orientations through the ventricular wall in a manner resembling a Japanese fan, observed in the geodesical arrangement, viewed with a 1 mm sampler corresponds to high RI values. At the post-neonatal period, the high RI areas interspersed with low RI in the complex way that blurs the geodesical pattern and creates inhomogeneities in the ventricular walls. There are resemblances between the topological pattern of high RI areas and the “cleavage planes” described by Torrent-Guasp who practised hand dissection. This tends to vindicate Torrent Guasp allegations of a reproducible post-natal pattern. But, the terms “cleavage plane” as used by Torrent Guasp have to be understood as the plane where he dissected the ventricular mass with his proper, although well methodologically defined, hand dissection technique. The present work sharpens Torrent Guasp’s insight, these Torrent Guasp’s “cleavage planes” are only architectural inhomogeneities in the ventricular mass, and are not, of course, real cleavage planes separating discrete muscular fascicles.

The terms “cleavage plane” have also be used in another meaning by Legrice [13] to describe the laminar organization of myocytes. Unfortunately, the present study cannot question this orthotropic laminar model because the present PLI method is unsuitable to visualize collagen and laminae but focuses instead on the principal orientation of myosin filaments.

This post-neonatal appearance of an inhomogeneous pattern in 3D myocardial cells architecture is a new result that has to be added to all the postnatal changes already documented from a hemodynamic, anatomical and histological point of view [14, 15, 19]. Until now, few studies were dedicated to the three-dimensional myocardial architecture modifications during this period in large mammals [16, 24]. These authors used diffusion tensor MRI (DTI) techniques and also showed postnatal change in fractional anisotropy. But presently, no relationship between their MRI FA and our PLI RI can be established, DTI deals with diffusion of water molecules while RI deals with myosin filament directions [16, 24].

This new result offers new perspectives to comprehend heart developmental remodelling. The change from the geodesic foetal fibre architecture to the post-neonatal inhomogeneous pattern is developmentally significant. As a matter of fact, a geodesic pathway generally results from an optimization process when there is no permanent obstacle to the reorientation of the principal orientation of the myocardial cells in the osculatory plane. Thus, only a limitation of reorientation ability combined with the drastic postnatal haemodynamic changes could lead to the new post-neonatal distribution of low- and high-RI layers described blurring the geodesical pattern. Post-natal increase of the collagen scaffold could be this obstacle to reorientation of myocardial cells. Data about developmental changes in heart collagen content in big animals are scarce; in pig, the collagen content and the cross-links increase significantly from birth to yearling piglet [15]. Unfortunately, our technique does not make it possible to study the collagen content neither qualitatively nor quantitatively. Thus, the role of this post-natal increase in the collagen weave network that would restrict the myocardial cells orientation in the osculatory plane, remains a conjecture.

The postnatal inhomogeneous 3D architecture we describe supports the observations of regional inhomogeneity of postnatal mechanical shortening and lengthening sequences in the LV wall, which result in a highly efficient overall function of the normal heart adaptive to the post-neonatal haemodynamic conditions [20]. The timing of these developmental changes specified in this paper is predictive of a postnatal modification of ventricular wall motions. The left ventricular twist must significantly increase during post-natal period. Fortunately, this conjecture can be checked by modern in vivo imaging studies of parietal movement changes after birth. We hope that specialists of speckle echocardiography would grasp the problem [8, 9].

## Conclusions

This study has shown a postnatal change in ventricular myocardial architecture, which became more inhomogeneous. The cell rearrangements responsible for the inhomogeneity in ventricular myocardial architecture are revealed by a variation of the regional isotropy index. These major changes are probably an adaptive consequence of the major haemodynamic changes occurring after birth during the neonatal period that generates major parietal stress variations and parietal remodelling.

## Electronic supplementary material

Below is the link to the electronic supplementary material.

Supplementary material 1 (AVI 772 KB)

Supplementary material 2 (AVI 694 KB)

Supplementary material 3 (AVI 2151 KB)

Supplementary material 4 (AVI 1895 KB)

Supplementary material 5 (DOCX 4 KB)
