# Supplementary material for: Postnatal myocardium remodelling generates inhomogeneity in the architecture of the ventricular mass
Source: Surg Radiol Anat. 2017 Nov 28;40(1):75–83. doi: 10.1007/s00276-017-1945-5 (PMC5820407; doi:10.1007/s00276-017-1945-5)
Supplement: Supplementary file 5 — Supplementary material 5 (DOCX 4 KB) [file 276_2017_1945_MOESM5_ESM.docx]

SUPPLEMENTARY MATERIAL LEGEND

Data supplement-1: Maps of LIC texture of a stillbirth human heart

Data supplement-2: Maps of RI of a stillbirth human heart

Data supplement-3: Maps of LIC texture of a 12 week post-natal human heart

Data supplement-4: Maps of RI of a 12 week post-natal human heart

**Legend of data supplement-1:**

Maps of LIC texture of a stillbirth human heart

The video presents the sequence of 30 texture images calculated every 1 from the apex to the base of the ventricles. The continuity of streamline textures from one section to another creates the illusion of flow motions.

**Legend of data supplement-2**:

Maps of RI of a stillbirth human heart, same heart as in data supplement 1

The video presents the sequence of 30 RI images (with LIC texture superimposed) calculated every 1 mm from the apex to the base of the ventricles. RI are shown in false colours from black, magenta for low RI values to yellow, orange for high RI values.

**Legend of** **data supplement-3**:

Maps of LIC texture of a 12 week post-natal human heart

The video presents the sequence of 52 LIC texture maps calculated every 1 from the apex to the base of the ventricles. The continuity of streamline textures from one section to another creates the illusion of flow motions. The marge of inferior left ventricle wall was abraded by one technical artefact.

**Legend of data supplement-4**:

Maps of RI of a 12 week post-natal human heart, same heart as in data supplement 1A

The video presents the sequence of 52 RI maps (with LIC texture superimposed) calculated every 1 mm from the apex to the base of the ventricles. RI are shown in false colours from black, magenta for low RI values to yellow, orange for high RI values.
